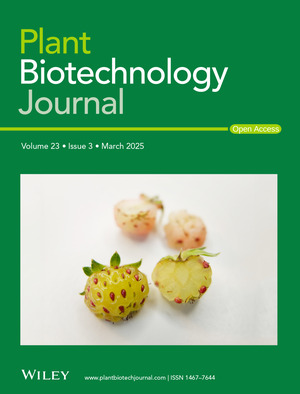# Issue Information

**DOI:** 10.1111/pbi.14389

**Published:** 2025-02-28

## Abstract

Front cover image:

Most strawberries accumulate anthocyanins in their epidermis and flesh, masking other important pigments as carotenoids. Therefore, despite their importance to plants and human nutrition, the mechanisms regulating natural variation of carotenoids in strawberry fruit remain largely unexplored. Amaya and colleagues reveal substantial variation using an interspecific F_2_ population and also diverse accessions from the cultivated strawberry *Fragaria × ananassa* and the beach strawberry *F. chiloensis*. Differential expression of the *FaCCD4* gene at chromosome 4B, encoding a carotenoid cleavage dioxygenase, is a mayor determinant of carotenoid accumulation and yellow flesh. The cover image features two fruits from distinct individuals of the interspecific F_2_ population. The fruit in the foreground, displaying yellow pigmentation, exhibits reduced *FaCCD4(4B)* activity, which results in enhanced carotenoid accumulation. Cover illustration refers to the article published in this issue (Amaya et al., pp. 679–691).